# A comprehensive study on the association of personal air pollution exposure with thyroid hormones in pregnant women

**DOI:** 10.1097/EE9.0000000000000495

**Published:** 2026-07-21

**Authors:** Sandra Rjeschni, Ariane Guilbert, Fanny Trecourt, Sarah Lyon-Caen, Sam Bayat, Anne Boudier, Patrice Faure, Benoît Chovelon, Christelle Corne, Anne Sophie Gauchez, Dorra Guergour, Rémy Slama, Claire Philippat, Johanna Lepeule

**Affiliations:** a Université Grenoble Alpes, INSERM U1209, CNRS UMR 5309, Institut pour l’Avancée des Biosciences (IAB), Team of Environmental Epidemiology Applied to Development and Respiratory Health, Grenoble, France; b Department of Pulmonology and Physiology, Grenoble University Hospital, La Tronche, France; c Synchrotron Radiation for Biomedicine Laboratory (STROBE), INSERM UA07, Grenoble Alpes University, Grenoble, France; d CHU Grenoble Alpes, Grenoble, France; e Service de Biochimie SB2TE, Institut de Biologie et Pathologie CHU Grenoble Alpes, Université Grenoble Alpes, Grenoble, France; f Département de Pharmacochimie Moleculaire, CNRS, UMR 5063, Université Grenoble Alpes, Grenoble, France.

**Keywords:** BTEX, Particulate matter, Nitrogen dioxide, Thyroxine, Personal exposure, Pregnancy, Thyroid

## Abstract

**Background:**

Over 10 epidemiological studies have investigated air pollutants’ association with thyroid hormone levels in pregnant women, but none have used personal air pollutant exposure data or included benzene, toluene, ethylbenzene, and xylene (BTEX). We assessed the relationship between personal exposure to nitrogen dioxide (NO_2_), particulate matter ≤2.5 µm (PM_2.5_), and BTEX with maternal thyroid hormones during pregnancy.

**Methods:**

The study relied on 437 pregnant women from the SEPAGES cohort (Grenoble, France, 2014–2017). Personal exposure to NO_2_, PM_2.5_, and BTEX was measured 1 week before blood sampling (mean: 19th gestational week), where thyroid hormones (free and total triiodothyronine (FT3, TT3), thyroxine (FT4, TT4), thyroid-stimulating hormone, and the TT3/TT4 ratio) were assessed. We linearly regressed air pollutants against individual thyroid hormone concentrations, adjusting for covariates, including urinary iodine concentration. Ambient NO_2_ and PM_2.5_ exposures were also analyzed for comparison.

**Results:**

Personal PM_2.5_ was positively associated with TT4 (β = 2.3%; 95% confidence interval [CI] = 0.4%, 4.2%, per doubling of exposure) and FT4 (β = 1.9%; 95% CI = −0.3%, 4.2%). On the contrary, negative associations between doubling the ethylbenzene exposure and TT4 (β = −1.2%; 95% CI = −2.4%, 0.1%) and FT4 levels (β = −1.3%; 95% CI = −2.7%, 0.1%) were observed. The association between ambient PM_2.5_ 1 week before hormone measurement and FT4 was consistent with the association observed for personal exposure. Associations between ambient PM_2.5_ and TT4 were positive but attenuated across all examined time windows.

**Conclusions:**

Personal short-term exposure estimates provide evidence of a potential association between PM_2.5_, ethylbenzene, and altered thyroxine (TT4 and FT4) levels in pregnant women, warranting replication in other populations and larger samples.

**What this study adds**

Our study is novel as it is the first to use personal air sensors for studying the short-term effect of air pollution on maternal thyroid hormones during pregnancy. We conducted a comparative analysis using personal and ambient PM_2.5_ and NO_2_ exposure levels and further considered the influence of BTEX compounds, which other studies have not investigated until now.

## Introduction

The Global Burden of Disease Report from 2019 reported that air pollution was the fourth risk factor for human-attributable mortality, both for men and women.^1^ This includes particulate matter (PM), especially PM with an aerodynamic diameter of 2.5 µm (PM_2.5_), which consists primarily of organic matter, black carbon, sulfate (SO_4_2^−^), nitrate (NO_3_^−^), ammonium (NH_4_^+^), and soil dust. PM_2.5_ can enter the bloodstream, and in pregnant women, the adsorbed PM_2.5_ components can cross the placental barrier to reach the fetus.^2^ An increasing number of studies reported an association between PM_2.5_ and NO_2_ exposure during pregnancy with slower fetal growth,^3^ including effects on birth weight,^4^ fetal measurements,^5^ small for gestational age,^6^ fetal death and dysplasia,^7^ and neurodevelopmental disorders.^8,9^

The mechanisms behind the associations between air pollution exposure during pregnancy and child health are not well understood. Disruption of the hormonal balance might be one of them. Maternal thyroid hormones (THs) are indeed crucial for embryogenesis, fetal growth, and development. Because the fetal thyroid gland begins to develop around 10 weeks of gestation and is functionally mature at around 18–20 weeks,^10^ before this period, the fetus is dependent on the mother’s TH production.

Animal studies showed that PM_2.5_ exposure interferes with TH regulation by affecting TH receptor levels and lowering thyroid peroxidase (TPO) levels, leading to decreased iodine oxidation and TH synthesis.^11–13^ Additionally, PM can promote oxidative stress and inflammatory responses that impair thyroid function, particularly by inhibiting iodine oxidation and TH biosynthesis, as found in a rat experiment.^11,14–17^ Finally, PM_2.5_ can also affect liver function by reducing the expression of transthyretin protein, impairing the transport and homeostasis of TH.^11,16,18^ In recent years, more than 10 epidemiological studies have investigated the association between air pollution and maternal TH during pregnancy.^2,12,16,19–26^ However, all published studies have relied on ambient exposures, which are prone to measurement error as they do not account for all microenvironments of the participants, and none have considered BTEX (benzene, toluene, ethylbenzene, para-, meta-, and ortho-xylenes). BTEX are organic volatile compounds widely used in fuel formulations and in a variety of industrial and consumer products, including adhesives, coatings, degreasers, detergents, paints, and pesticides. As a result, exposure levels may be higher in indoor environments than in outdoor settings.^27^ BTEX are highly toxic and pathogenic.^27,28^ Earlier research showed that TH regulation and function in adults and neonates may be impacted by exposure to active and passive cigarette smoke, which contains BTEX.^29^ Some animal studies generally conclude that there is an association between BTEX and circulating TH concentrations, but their findings are inconsistent.^30–32^

In the current study, we addressed these gaps by studying the association of short-term personal exposure to NO_2_, PM_2.5_, and BTEX with the levels of maternal TH during pregnancy.

## Methods

### Study population

The study uses data from the SEPAGES (“Suivi de l’Exposition à la Pollution Atmosphérique durant la Grossesse et Effet sur la Santé”) cohort in Grenoble, France. After the recruitment period from July 2014 to July 2017, 484 pregnant women participated in the study. They had to meet the following conditions: being 18 or older, less than or equal to 18 weeks pregnant at the start of the study, having a singleton pregnancy, living in the study area, planning to give birth in one of the four maternity hospitals in the Grenoble area, participating in interviews, completing questionnaires, and undergoing clinical examinations.^33^

Of the 484 participants, 437 women were included in our analyses, excluding those without any air pollution and thyroid hormone measurement and those taking medications for thyroid-related conditions.^34^

Informed written consent was obtained from the participants before inclusion. The study was approved by the Ethics Committee Sud-Est V and the National Commission on Informatics and Liberty.

### Assessment of air pollution exposure

#### Personal exposure

Each woman was given a device to measure personal exposure to PM (MicroPEM active air sampler, RTI, Research Triangle Park, NC), NO_2_ (passive sampler from Passam AG, Männedorf, Switzerland), and BTEX (passive sampler from Passam AG, Männedorf, Switzerland) for a 1-week period (7.9 ± 0.4 days) for one to three times during pregnancy. The 7-day window was chosen to balance feasibility and observance, considering the practicality for participants and the relatively short half-life (1 day for 3, 5, and 3-triiodothyronine, 5–7 days for thyroxine) of the hormones. Participants were advised to keep the devices on throughout the measurement week and to ensure the devices remained nearby (within 2 m) when removal was necessary, such as during sleep or bathing. The MicroPEM device provides high-quality PM_2.5_ data by recording both real-time mass concentration and collecting particulate samples on a 25-mm polytetrafluoroethylene filter for gold-standard gravimetric analysis. To ensure data accuracy, filters were pre- and post-weighed using a microbalance in a temperature- and humidity-controlled environment. Additionally, real-time measurements were calibrated against gravimetric filter data using MicroPEM Docking Station software, with instruments undergoing calibration before each measurement period. Validation studies have shown that the MicroPEM performs well in different weather and air pollution conditions, with R2 coefficients higher than 0.8 between the MicroPEM data and measurements from PM_2.5_ fixed stations (considering either real-time or gravimetric concentrations).^35^

For our analyses, we used the mean air pollutant concentrations corresponding to the week preceding the blood draw used for thyroid hormone assessment. So, 429 participants for NO_2_, 305 for PM_2.5_, and 422 for BTEX were included in the analyses.^33^ Of note, the MicroPEM active air sampler used to measure personal PM_2.5_ was introduced later than the other sensors, which explains why fewer participants had personal PM_2.5_ measurements compared with NO_2_ or BTEX.

### Measurements of thyroid hormone, selenium, and iodine concentrations

Skilled SEPAGES fieldworkers took nonfasting maternal blood samples at the participants’ residences. For the majority of the participants, sampling occurred during the second-trimester study visit, whereas 13.7% were sampled around the 27th gestational week due to a missed second-trimester visit. After collection, samples were brought on ice to the Grenoble University Hospital’s biobank, where the blood underwent processing procedures, after which serum aliquots were stored at −80 °C. The serum concentrations of protein-bound and free thyroxine (T4) and 3, 5, and 3-triiodothyronine (T3) were quantified utilizing the RIA-Gnost kit from CisBio Bioassays. The total T3 (TT3) and T4 (TT4) were derived from the summation of the free and protein-bound concentrations (FT3, FT4). Subsequently, the TT3 to TT4 ratio (TT3/TT4) was calculated, which indicates TT4 deiodination into the bioactive TT3 form. Thyroid-stimulating hormone (TSH) was quantified in maternal sera using LOCI Chemiluminescence on a Dimension Vista analyzer from Siemens.^36^ Measurement of both free and total T3 and T4 allowed determination of whether air pollutants affect overall thyroid hormone levels or just the free, active forms available for immediate use by the body. Additionally, selenium was quantified in maternal sera utilizing inductively coupled plasma mass spectrometry.^37^ Selenium is an essential micronutrient required for the biosynthesis of selenoproteins involved in the peripheral conversion of FT4 to FT3, and it is also a vital antioxidant in the thyroid gland.

Iodine is essential for producing TH, but it cannot be produced by the human body; its deficiency is still widespread in many regions of Europe, including France. Quantification was performed on a pool of three urine samples collected over a day in the second trimester, using inductively coupled plasma mass spectrometry.^38^

### Statistical analyses

To examine the relationships between each air pollutant personal exposure (NO_2_, PM_2.5_, benzene, toluene, ethylbenzene, p-xylene, m-xylene, o-xylene) and each TH (TSH, FT3, TT3, FT4, TT4, TSH) or ratio (TT3/TT4), we used adjusted linear regression.

TH, as well as the ratio TT3/TT4 and BTEX, were ln-transformed to reduce the impact of extreme values. Personal NO_2_ and PM_2.5_ were also ln-transformed to facilitate comparison of effect estimates. Diagnostic plots were consistent with those from the non-transformed data.

Based on literature and biological plausibility, the models were adjusted for a priori selected confounders and predictors of TH, including education level (≤2 years after high school, 3–4 years after high school, ≥5 years after high school [reference]), maternal smoking during the first trimester of pregnancy (yes [reference], no, smoking status unknown), parity (nulliparous [reference] and multiparous), maternal age (continuous, linear and quadratic terms), ambient temperature during sensor wearing (in °C, continuous).^39^ Pre-pregnancy body mass index (BMI, kilograms per square meter, continuous), gestational age at serum collection (continuous, weeks), time of blood collection ([0, reference] ≤ 8h30, (1) ≤13h, (2) ≤18h, (3) >18h), maternal urinary iodine concentrations (ln-transformed), and the analytical batch (categorical variable with six to seven categories depending on the hormones) for all hormones (except TSH, for which no batch effect was observed) were also included. Missing values on covariates, except maternal smoking (coded as unknown status), were singly imputed by the median or mode (n = 4 for pre-pregnancy BMI, n = 1 for maternal education).

Using penalized spline models in the generalized additive models framework, we examined the shape of the relationship between ambient temperature and TH and between ln-transformed personal air pollutants and TH. The spline analysis only showed a potentially nonlinear effect for the PM_2.5_–FT4 association, but with a borderline *P* value of 0.04, which was influenced by rare values at the ends of the spline (Figure S2, https://links.lww.com/EE/A439). We thus assumed a linear dependence for all associations tested and included personal exposure as a continuous variable in our regression models.

Results are presented as percentage changes in outcomes for each doubling of air pollution exposure. The initial betas derived from the linear regression model were converted to percentage changes using the formula (2^β^ − 1) × 100.

Main analyses were not adjusted for multiple testing, as this study does not test a single omnibus null hypothesis but evaluates each association independently.^40^ Moreover, multiple-testing corrections could increase type 2 errors and obscure meaningful findings,^41–43^ making studies overly conservative and potentially masking important associations, particularly in exploratory, hypothesis-generating research such as this first study based on personal exposure.

In the analyses, we used the R programming language (version 4.2, R Foundation for Statistical Computing, Vienna, Austria) and the lm() function for the regression models.

### Sensitivity analyses

The following sensitivity analyses were performed: (1) excluding participants with unknown smoking status, (2) excluding influential values with a studentized residual greater than 3 in absolute value (*N* ranged between 294 and 420 depending on the model), (3) including selenium as a covariate, (4) excluding women who smoked during the first trimester, (5) excluding women with extreme BTEX values (benzene >22 µg/m^3^, toluene >75 µg/m^3^, ethylbenzene >31 µg/m^3^, p-xylene >20 µg/m^3^, m-xylene >40 µg/m^3^, o-xylene >24 µg/m^3^), (6) excluding women with a blood measurement occurring in the third trimester, (7) correcting main analyses for multiple testing using the Benjamini–Hochberg procedure.

### Additional analyses

The main goal of the paper is to explore the association between personal air pollution and maternal TH. However, as the existing literature has only used ambient exposure to NO_2_ and PM_2.5_ to investigate the association with TH levels, we conducted additional analyses where we investigated the association of maternal TH with ambient NO_2,_ and PM_2.5_ estimates at the participants’ home addresses: (1) during the same period when women wore the personal sensors, (2) in the 12 weeks before the blood measurement, and (3) from the time of conception until the blood measurement. These ambient estimates relied on a dispersion model (including data on emission, meteorology, and permanent monitoring stations) developed by the regional air pollution monitoring network, Atmo Auvergne-Rhône-Alpes, at fine spatial (10 m) and temporal (hourly) resolution.^44,45^ We did not ln-transform ambient exposures, as their distributions were already normal and previous studies did not perform such a transformation. The effect estimates represent the % change in outcomes when increasing the exposure level by 10 µg/m^3^. To compare the results of these additional analyses with our main analyses, we restricted the study population to participants with both personal and modeled air pollution estimates available (N = 298 for PM_2.5_ and 419 for NO_2_). This restriction was necessary to ensure comparability and avoid misleading conclusions due to differences in sample sizes. Consequently, we excluded 10 participants for missing ambient NO_2_ and seven for missing ambient PM_2.5_ exposure data.

## RESULTS

### Characteristics of the study participants

Among included women, 56% had completed 5 years or more of education beyond high school, and only 5.3% reported smoking during the first trimester of pregnancy, while nearly 9% had an unknown smoking status (Table 1). Over half of the women (54%) already had one or more children, with an average age of 32.1 years and a pre-pregnancy BMI of 21.5 kg/m^2^.

**Table 1.** Characteristics of the pregnant women included in this study, n=437, SEPAGES cohort, recruitment period 2014–2017

Characteristic	Category	N (%)/ median (Q1, Q3)
Education level	≥5 yr after high school	245 (56%)
	3–4 yr after high school	116 (27%)
	≤2 yr after high school	75 (17%)
	Missing	1 (0.2%)
Parity	Nulliparous	199 (46%)
	Multiparous	238 (54%)
Maternal tobacco consumption (during first trimester)	Not smoking	376 (86%)
	Smoking	23 (5.3%)
	Unknown status	38 (8.7%)
Time of blood collection	Before 8.30 am	39 (8.9%)
	Between 8.30 am and 1 pm	170 (39%)
	Between 1 pm and 6 pm	137 (31%)
	After 6 pm	91 (21%)
Gestational age at blood collection (in wk)		19.1 (17.9, 20.4)
Maternal age (in yr)		32.1 (29.9, 35.2)
Maternal pre-pregnancy BMI (in kg/m^2^)		21.5 (19.8, 23.9)
	Missing	4 (1%)
Ambient temperature during air pollution measurement sensor wearing (in °C)		12 (6, 19)

The median gestational age at blood sampling was 19.1 gestational weeks, and blood collection occurred less frequently before 8:30 am. The urinary iodine, serum selenium, TH concentrations, and air pollutant exposure levels are described in Table 2. A large percentage of the women (80%) had urinary iodine below the World Health Organization (WHO) threshold. Women were exposed on average to 20.9 µg/m^3^ (standard deviation [SD] = ±7.9 µg/m^3^) of NO_2_ and 13.8 µg/m^3^ (SD = ±7.1 µg/m^3^) of PM_2.5_ as estimated by the personal sensors. The estimated ambient PM_2.5_ exposure was similar (14.1 µg/m^3^ [SD = ±8.5 µg/m^3^]), whereas the ambient average NO_2_ was higher (26.0 µg/m^3^ [SD = ±15.3 µg/m^3^]) than the personal exposure during the same period of 1 week.

**Table 2.** Summary of thyroid hormone, iodine, selenium, and air pollution exposure levels

Variable	N	Mean	SD	Min.	Q1	Median	Q3	Max.	IQR	In range^a^	<Range^a^	>Range^a^
Biological markers												
FT3 (in pg/mL)	437	2.1	0.3	1.5	1.9	2.1	2.3	3.4	0.4	324 (74.1%)	113 (25.9%)	
TT3 (in ng/mL)	405	1.2	0.3	0.7	1.0	1.2	1.3	2.4	0.3	398 (98.3%)		7 (1.7%)
FT4 (in pg/mL)	435	7.5	1.5	4.1	6.4	7.2	8.4	13.4	2.0	259 (59.5%)	176 (40.5%)	
TT4 (in ng/mL)	437	96.9	16.6	54.5	85.4	95.6	105.4	230.4	20.0	364 (83.3%)		73 (16.7%)
TSH (in mUI/L)	437	1.4	0.7	0.0	0.9	1.3	1.8	4.5	0.9	414 (94.7%)	13 (3.0%)	10 (2.3%)
TT3/TT4	405	0.013	0.003	0.006	0.011	0.012	0.014	0.025	0.003			
Urinary iodine (in µg/L)	437	113.1	93.0	12.9	56.7	89.3	134.5	941.6	77.8	61 (14.0%)	350 (80.1%)	26 (5.9%)
Serum selenium (in µmol/L)	364	1.0	0.1	0.7	0.9	1.0	1.1	1.5	0.2			
Exposure levels^b^												
NO_2_ (personal) (in µg/m^3^)	429	20.9	7.9	5.3	15.8	20.0	24.7	85.0	8.9	325 (75.8%)	-	104 (24.2%)
NO_2_ (ambient) (in µg/m^3^)	419	26.0	15.3	0.4	14.8	23.9	34.8	81.4	20.0	228 (54.4%)	-	191 (45.6%)
PM_2.5_ (personal) (in µg/m^3^)	305	13.8	7.1	1.5	9.2	12.9	18.0	51.1	8.8	195 (63.9%)	-	110 (36.1%)
PM_2.5_ (ambient) (in µg/m^3^)	298	14.1	8.5	3.9	8.6	11.1	17.7	49.6	9.1	209 (70.1%)	-	89 (29.9%)
Benzene (in µg/m^3^)	422	2.2	6.9	0.3	0.8	1.3	2.2	131.8	1.4			
Toluene (in µg/m^3^)	422	8.4	13.6	0.3	3.3	4.8	7.4	168.2	4.1			
Ethylbenzene (in µg/m^3^)	422	2.6	4.5	0.3	1.1	1.6	2.4	49.3	1.3			
P-Xylene (in µg/m^3^)	422	3.1	4.4	0.3	1.4	2.0	3.0	40.9	1.6			
M-Xylene (in µg/m^3^)	422	4.3	8.1	0.3	1.4	2.1	3.7	101.7	2.3			
O-Xylene (in µg/m^3^)	422	2.1	3.3	0.3	0.9	1.2	1.8	35.0	1.0			

a Reference ranges used: Free thyroxin FT4 (7–18 pg/mL), Total thyroxin TT4 (45–110 ng/mL), Free triiodothyronine FT3 (2–4.3 pg/mL), Total triiodothyronine TT3 (0.6–1.9 ng/mL), Thyroid-stimulating hormone TSH during first trimester (0.358–2.5 mUI/L) and second/third trimester (0.358–3 mUI/L) given by the French Haute Autorité de Santé. Urinary iodine concentration (150–249 µg/L) provided by the WHO guidelines^46^. Daily ambient air quality recommendation for particular matter with a diameter smaller or equal to 2.5 µm PM_2.5_ (<15 µg/m^3^) and nitrogen dioxide NO_2_ (<25 µg/m^3^) according to the WHO guidelines.^47^

b Measured during the week preceding blood sampling.

IQR indicates interquartile range; Max., maximal value; Min., minimal value; N, number of participants; SD, standard deviation; Q1, 25% quantile; Q3, 75% quantile.

Personal and ambient PM_2.5_ and NO_2_ had Spearman correlation factors of 0.36 and 0.53, respectively. In contrast, ambient PM_2.5_ and ambient NO_2_ were highly correlated with a Spearman correlation factor of 0.78 (see Figure S1a https://links.lww.com/EE/A439.

Participants with and without personal PM_2.5_ measurement were similar across most characteristics (Table S4 https://links.lww.com/EE/A439). However, differences were observed in gestational age at blood collection and education level. Indeed, participants with personal PM_2.5_ data had blood collected at a lower gestational age compared with those without personal PM_2.5_ data and were more likely to have an intermediate level of education.

The personal exposure levels for the different BTEX compounds were around 2.1–4.3 µg/m^3^ with an SD between 3.3 and 8.1 µg/m^3^, except for toluene, whose mean exposure was higher: 8.4 µg/m^3^ (SD = ±13.6 µg/m^3^).

### Association of air pollution exposure and maternal hormone levels

#### Main analyses—FT4

**Figure 1.**
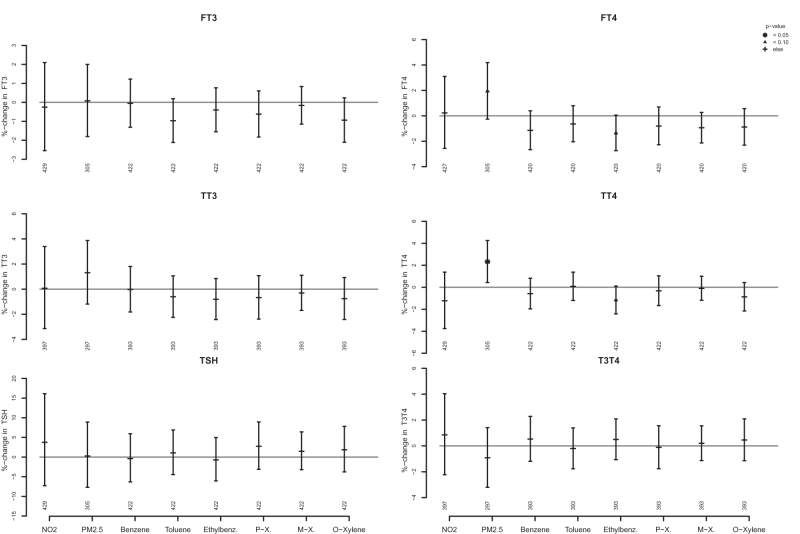
Adjusted associations between air pollution exposure and thyroid hormones in the SEPAGES cohort. Effect estimates represent % change in outcomes when doubling the exposure level and the 95% CI. Thyroid hormones, as well as pollutants, were ln-transformed. % changes were calculated from the original betas using the formula: (2^β^ − 1) × 100. A list of the β values, the 95% CI values, can be found in Table S1 https://links.lww.com/EE/A439. Analyses were adjusted for maternal age, pre-pregnancy BMI, education level, maternal smoking during the first trimester of pregnancy, ambient temperature during sensor wearing, parity, gestational age at blood collection, time of blood collection, maternal urinary iodine concentrations, and the analytical batches for each specific hormone except TSH. Overall, n = 437, but for each analysis, refer to the particular numbers printed in the plot under each CI, SEPAGES cohort, and recruitment period 2014–2017.

Doubling the level of PM_2.5_ showed a positive association with FT4 (β = 1.94%; 95% confidence interval [CI] = −0.26%, 4.19%), while doubling the level of ethylbenzene showed a negative association with FT4 concentration (β = −1.35%; 95% CI = −2.74%, 0.06%) (Figure 1 and Table S1 https://links.lww.com/EE/A439). Consistent negative associations were observed with benzene and m-xylene exposures.

#### Main analyses—TT4

PM_2.5_ was positively associated with TT4 (β = 2.32%; 95% CI = 0.43%, 4.25%), while ethylbenzene was negatively associated with TT4 concentration (β = 1.16%; 95% CI = −2.42%, 0.11%). In the main analyses, no other exposures were associated with this hormone.

#### Main analyses—other thyroid parameters

We did not observe any association of FT3, TT3, TSH, and the T3/T4 ratio with personal air pollutant exposure.

#### Sensitivity analyses—personal exposure

Sensitivity analyses were generally consistent with the main results (Figure S3 https://links.lww.com/EE/A439; Table S1 https://links.lww.com/EE/A439). Modest attenuations toward the null were observed for: PM_2.5_–FT4 association when excluding third-trimester blood assessments; ethylbenzene–FT4 association when excluding third-trimester blood assessments, excluding high ethylbenzene values, or adjusting for selenium; PM_2.5_–TT4 association when excluding high residuals; ethylbenzene–TT4 association when excluding participants with extreme BTEX values, high residuals or third-trimester blood assessments; including selenium. After correction for multiple testing, no associations were statistically significant (Table S3 https://links.lww.com/EE/A439).

#### Additional analyses—ambient exposure

**Figure 2.**
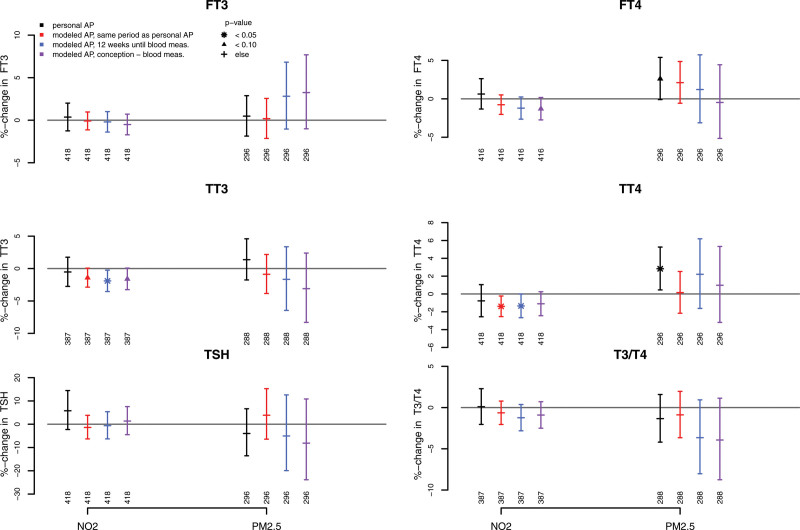
Comparison of adjusted associations between ambient and personal air pollution exposure and thyroid hormones in the SEPAGES cohort. Effect estimates represent %change in outcomes when increasing the exposure level by 10 µg/m^3^ and 95% CI. Thyroid hormones were ln-transformed, but the exposure was not. %changes were calculated from the original betas using the formula: (exp(10 × β) – 1) × 100. In black: personal air pollution exposure one week preceding the TH blood draw; in red: ambient air pollution exposure 1 week preceding the TH blood draw; in blue: ambient air pollution exposure 12 weeks preceding the TH blood draw; in purple: ambient air pollution exposure from conception to the week of the blood draw. A list of the β values and the 95% CI values can be found in Table S2 https://links.lww.com/EE/A439. Analyses were adjusted for maternal age, pre-pregnancy BMI, education level, maternal smoking during the first trimester of pregnancy, ambient temperature during sensor wearing, parity, gestational age at blood collection, time of blood collection, maternal urinary iodine concentrations, and the analytical batches for each specific hormone except TSH. Overall n < 437, but for each analysis, refer to the numbers printed in the plot under each CI; SEPAGES cohort; recruitment period 2014–2017. AP indicates air pollution.

To further inform the comparison of our results with previous studies, which all focused on ambient exposure, we also conducted additional analyses using ambient exposures (rather than personal exposures) and considering longer exposure windows (Figure 2 and Table S2 https://links.lww.com/EE/A439). Regarding FT4, the association with ambient PM_2.5_ in the week before the hormone measurement was similar to the association with personal PM_2.5_ exposure, although the effect size was smaller. The associations attenuated toward the null when using ambient PM_2.5_ measured over longer time intervals (the 12 weeks before hormone measurement or from conception to hormone measurement). The positive association observed between personal PM_2.5_ and TT4 was attenuated when considering ambient PM_2.5_.

Ambient NO_2_ exposure was associated with lower TT3 (in the 12 weeks before hormone measurement) and TT4 (for 1 or the 12 weeks before hormone measurement), while no association was found with personal NO_2_ exposure.

## DISCUSSION

**Figure 3.**
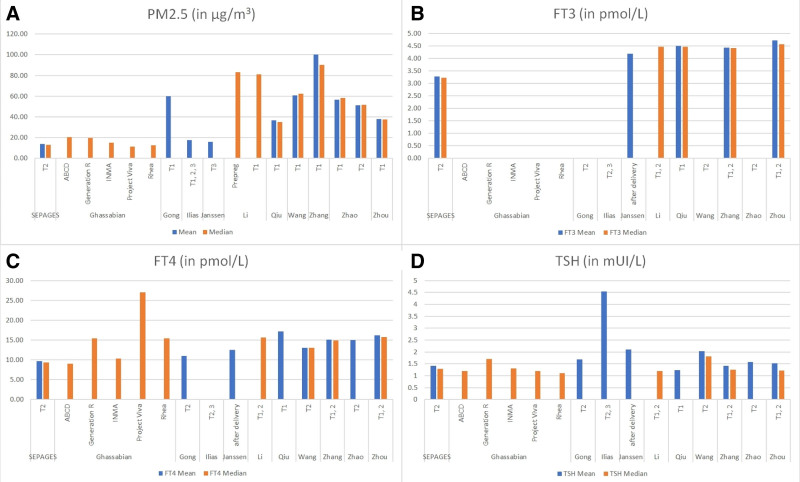
Comparison across studies of the mean/median of (A) PM_2.5_ (personal exposure in SEPAGES vs. ambient in other studies), (B) FT3, (C) FT4, and (D) TSH concentrations.^2,12,19–26^ Using as conversion factor FT3: 1 pg/mL = 1.5361 pmol/L, FT4: 1 ng/dL = 12.872 pmol/L.

This study investigated for the first time the effects of personal exposure to BTEX, PM_2.5_, and NO_2_ on the maternal level of TH during pregnancy. We identified positive associations of PM_2.5_ with TT4 and FT4, along with negative associations between ethylbenzene and the same hormones. When considering ambient exposure, ambient PM_2.5_ in the week before the hormone measurement showed a similar positive association with FT4, while ambient NO_2_ 1 or 12 weeks before hormone measurement was negatively associated with TT3 and TT4. Research on the impact of air pollution on maternal TH, such as FT3, FT4, and TSH, has recently gained significant attention.^2,12,16,19–26^ To facilitate comparison, Figure 3 shows the mean and median ambient air pollution exposure and hormone levels from 10 papers published between 2017 and 2023, while Table 3 synthesizes the existing literature.

**Table 3:**
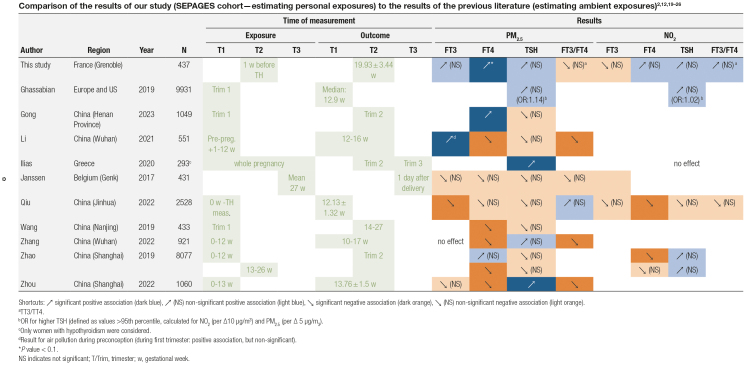
Comparison of the results of our study (SEPAGES cohort—estimating personal exposures) to the results of the previous literature (estimating ambient exposures)^2,12,19–26^

### Association of PM_2.5_ with TH levels

This study detected a positive association between personal PM_2.5_ and TT4 as well as between PM_2.5_ and FT4. The ef-fect on FT4 was consistent when using ambient PM_2.5_ in SEPAGES during the same period as personal PM_2.5_. Still, it attenuated toward the null when using longer air pollution exposure intervals. The present results tend to contrast with prevailing literature. Remarkably, five studies concluded that exposure to ambient PM_2.5_ in the first^2,12,24,26^ and the second trimesters^25^ of pregnancy results in a decrease in maternal FT4. At the same time, Qiu^23^ (in the first trimester), Zhao^25^ (in the first trimester), and Janssen^22^ (in the third trimester) did not find associations, even though Qiu^23^ and Janssen^22^ found a negative trend between FT4 and PM_2.5_. The study by Ghassabian^19^ encompassed four European cohorts and one US cohort, which is particularly interesting for comparative purposes since the European cohorts share more similar exposure levels. However, this study did not directly investigate TH; instead, it examined hypothyroxinemia (low FT4 but normal TSH levels). It found that exposure to PM_2.5_ during the first trimester was associated with an increased likelihood of hypothyroxinemia, which tends to contradict our results. Only Gong^20^ obtained similar results to ours. The influence of air pollutants on TT3 and TT4 has not been examined in any of the previous studies.

For FT3, we did not find any association with personal or ambient PM_2.5_ exposure, which is in line with previous reports,^22,24,26^ while negative^23^ and positive^12^ associations have also been reported with first-trimester ambient exposure. Zhou,^26^ as well as Ilias,^21^ observed a positive association between PM_2.5_ and TSH, but Gong,^20^ Janssen,^22^ Qiu,^23^ Zhang,^24^ Zhao,^25^ Li,^12^ and Wang^2^ did not find associations, as we did. The association between PM_2.5_ and FT4/FT3 was negative for Zhou,^26^ Zhang,^24^ and Li,^12^ while our study did not observe an association between PM_2.5_ and TT3/TT4.

### Association of NO_2_ with TH levels

In SEPAGES, the present results showed negative relationships between ambient NO_2_ exposure (but not with personal exposure) and TT3 and TT4. Associations were more specifically observed for exposure windows of 1 or 12 weeks before blood measurement. One possible reason for the stronger effect of ambient NO_2_ exposure could be that NO_2_ sources, such as traffic, are predominantly outdoors, while personal sources like gas stoves are less common, with average ambient NO_2_ levels also being higher than personal exposure (26 ± 15.3 µg/m^3^ vs. 20.9 ± 7.9 µg/m^3^). Only three papers studied ambient NO_2_ exposure in relation to TH levels, and only one compared it with the FT4/FT3 ratio. Zhao^25^ examined the effect of NO_2_ exposure during the first and second trimesters on FT4 and TSH levels. He found that NO_2_ exposure during the first trimester was negatively associated with FT4 (Table 3). Qiu^23^ studied the effect of NO_2_ exposure from the last menstrual period until TH measurement on FT3, FT4, TSH, and FT4/FT3. He only reported a negative association between NO_2_ and FT4. Ilias,^21^ who measured the NO_2_ level during the whole pregnancy, found no association between NO_2_ and TSH.

### Association of BTEX with TH levels

We could not find any human or animal study that explored the influence of gestational BTEX exposure on TH. Five animal studies on adult rabbits, rats, mice, and birds examined benzene, toluene, and xylene (BTX) exposure over 1 week to 1 month.^30–32,48,49^ These studies reported varied results regarding the association between BTX exposure and TH. For instance, Fernie^30^ found that BTX is negatively associated with T4, Ketan^31^ found positive associations with T3 and T4, while others did not observe effects.^48^ None of them investigated ethylbenzene, the compound for which we found negative associations with TT4 and FT4.

### Differences across studies

Our study reports positive associations of PM_2.5_ with FT4 and TT4, whereas most previous studies (including large cohorts from China and Europe) have reported negative associations. Several reasons may explain these differences. First, whereas previous studies relied on ambient air pollution measurements, we used personal exposure measurements. Ambient pollutant levels focus on outdoor levels and are thus less representative of personal levels, accounting for the different microenvironments. This distinction was evident in our study when comparing personal and ambient exposures in relation to TH, because associations observed with personal indicators were not consistently replicated using ambient indicators. Second, additional analyses using ambient exposures showed that the observed associations also depend on the exposure time window considered. In the SEPAGES study, blood was mainly collected around the 19th week of pregnancy, with a few exceptions for which blood was collected around the 27th week of pregnancy (13.7%) (see Table 1). In past literature, blood sampling timing varied from the first trimester up to delivery,^22,24^ with a likely effect on TH levels.^50^ Also, most of the previous studies did not consider short-term exposure to air pollution (7 days in our study) but longer periods (mostly one trimester). Short-term exposure to air pollution may affect biological mechanisms differently, resulting in distinct consequences on TH levels compared with prolonged exposure scenarios. In the literature, there is currently no agreement on critical windows of exposure linking prenatal exposure to air pollution and TH.^51^ Third, exposure levels were very different across studies. Of the 10 published studies, seven were conducted in China, where PM_2.5_ exposure levels were notably around double those in the European studies (with Ghassabian also including data from the United States) (see Figure 3a). Differences in PM_2.5_ composition may also explain the different associations observed with TH levels, suggesting that the impact may depend on PM individual constituents rather than overall PM_2.5_ levels. Fourth, studies showed different TH levels across populations. In SEPAGES, the average FT4 level was 9.62 pmol/L (7.5 pg/mL), falling at the lower end of the recommended range (9.01–23.17 pmol/L),^34,52^ while the other studies, except Gong^20^ and the ABCD and INMA studies in Ghassabian,^19^ reported higher FT4 values (Figure 3c). The mean FT3 level of SEPAGES was 3.28 pmol/L (2.1 pg/mL), at the lower end of the recommended range (3.07–6.61 pmol/L), whereas the other studies tended to have FT3 levels in the middle of the recommended range (see Figure 3b).^34,52^ Our TSH levels align closely with the previous studies (see Figure 3d), except for Ilias,^21^ who observed higher average TSH levels in pregnant women with hypothyroidism. The majority of our study population had urinary iodine levels below the WHO threshold. Although iodine level was not reported in previous studies, worldwide data suggest that the prevalence of iodine deficiency is higher in Europe than in Southeast Asia.^46^ Given that iodine is essential for the production of TH, this may explain the result discrepancies across studies from different locations.

### Mechanistic hypotheses

The mechanisms by which air pollutants might influence maternal TH concentrations, particularly TT4, FT4, and TT3, remain unclear. Gondou,^47^ Li,^12^ and Mannisto^53^ proposed that PM_2.5_ may increase the activity of type 2 deiodinase, thus accelerating the conversion of T4 to T3, resulting in decreased FT4 levels, as observed in several other previous studies.^2,12,24–26^ On the other hand, the literature suggests that the production of reactive oxygen species could be triggered by PM exposure and its detached components, leading to oxidative stress, a concept supported by Dong,^11^ Zhou,^26^ and Wang.^2^ According to Riggs,^54^ increased oxidative stress and inflammation levels post-PM_2.5_ exposure might activate the hypothalamic–pituitary–thyroid axis, which governs, among others, TH production. Notably, PM_2.5_ proved to interfere with TPO, impairing iodide oxidation and thyroglobulin iodination, and therefore reducing circulating thyroid hormone concentrations in animal models.^11^ In a Belgian cohort, placental iodine mediated part of the relationship between ambient PM_2.5_ exposure and cord blood FT4 concentrations.^55^ In a study conducted in early pregnancy in the American MADRES cohort, the iodine status modified the relationship between ambient PM and serum TSH in pregnant women.^56^

### Strengths

In the described articles, the researchers used ambient NO_2_ and PM estimates at the residential addresses of the women, which were approximated by exposure models relying on fixed monitoring station data, satellite data, or both. These estimates are prone to measurement error, as they do not consider factors like a woman’s frequent travel or spending extended time indoors. An innovation in our study is using personal exposure assessment while comparing results with ambient exposure estimates. Although only Zhou^26^ measured personal air pollution levels using sensors in a subset of participants (N = 329), finding that personal PM_2.5_ values were higher than ambient estimates (49.70 vs. 44.77µg/m^3^), they did not use these data for statistical analysis.

Besides, our analysis includes iodine as a covariate, which has not been included in the other studies and was mentioned as a limitation, as iodine is an essential component of thyroid hormone production; insufficient iodine can significantly influence thyroid function and hormone production.

### Limitations

By relying on personal exposure measurements and objective measures of hormones, we sought to maximize statistical power through minimized measurement error.^57–60^ Nonetheless, air pollution sensor deployment limited the sample size in the present study to that of,^2,12,21,22^ or smaller^19,23–26,47^ than, previous studies. Second, the study was restricted to the region of Grenoble, with a study population that was, on average, well-educated and older than the overall pregnant women in France.^33^ While studying a relatively homogeneous population may reduce residual confounding, it may prevent the extrapolation of our findings to populations characterized by lower socioeconomic status or higher levels of air pollution exposure. In particular, emerging evidence suggests that socioeconomic deprivation may modify the association between air pollution exposure and thyroid hormone levels.^56^ This highlights the need for future studies explicitly designed to assess effect modification by deprivation and related social determinants of health. Third, this study did not investigate the specific components of PM_2.5_, which may more specifically explain associations with TH. Given the complexity of PM_2.5_, investigating its specific components, as done by Zhou^26^ and Qiu,^23^ would have allowed for more precise identification of which particle components affect TH. Fourth, our protocol provided only cumulative one-week exposure, whereas daily exposure data would have enabled investigation of the temporal relationship between air pollution exposure and TH using distributed lag models, including the identification of lag-specific effects. Fifth, we relied on uni-pollutant models, which could not take into account confounding by co-pollutants and assess possible joint effects. This may especially affect the reliability of the associations observed for ethylbenzene, which exhibited strong correlations with other BTEX compounds. While mixture models may provide a more accurate and comprehensive identification of harmful pollutants, no single method seems able to robustly disentangle effects in the presence of highly correlated pollutants, exerting opposing effects, or when the signal is driven primarily by a single exposure.^61^ Lastly, even though we adjusted the model for confounders and TH concentration predictors, on which a major part of other studies adjusted, we cannot avoid residual confounding altogether. In particular, we only excluded participants who reported thyroid medication use, which is an incomplete proxy for disease status. Indeed, some participants with preexisting thyroid disorders may be nonadherent or intermittently adherent to medication and may have biased associations between air pollution and TH through residual confounding.

## CONCLUSION

Relying on personal air sensors, accounting for indoor and outdoor sources of exposure, we observed positive associations between PM_2.5_, TT4, and FT4, while previous studies have recorded negative associations with ambient exposure. Ethylbenzene showed negative associations with TT4 and FT4. Future studies relying on larger sample sizes and assessing whether these hormonal changes relate to child health outcomes are needed to confirm and expand on these findings.

## Conflict of interest statement

The authors declare that they have no conflicts of interest with regard to the content of this report.

## ACKNOWLEDGMENTS

SEPAGES biospecimens are stored at Grenoble University Hospital (CHU-GA) biobank (bb-0033-00069); we would like to thank the whole CRB team, led by Pr. P. Mossuz and Mr. P. Lorimier, and in particular the technicians for the huge work of biospecimens processing and pooling: Mrs. W. Jayar, Mrs. L. Than, Mrs. J. Lafond, Mrs. C. Chalazon, Mr. T. Caffaratti, Mrs. N. Francini, and Mr. G. Schummer. We thank the SEPAGES study group: A. Licinia (Groupe Hospitalier Mutualiste, Grenoble), S. Bayat, P. Hoffmann, E. Hullo, (Grenoble Alpes University Hospital, La Tronche), X. Morin (Clinique des Cèdres, Echirolles), A. Morlot (Clinique Belledonne, Saint-Martin d’Hères), J. Lepeule, S. Lyon-Caen, M.Ouidir, C. Philippat, I. Pin, J. Quentin, V. Siroux, and R. Slama (Grenoble Alpes University, Inserm, CNRS, IAB).

Many thanks to Dr. I. Pin, who was the SEPAGES PI from 2012 until 2022. We also thank Mr. Y. Gioria, Mrs. A. Guerrache-Siebert, clinical research assistants; Mrs. M. Marceau, nurses; Mrs. E. Charvet, midwives; Mrs. M. Graca, Mrs. K. Gridel, Mrs. C. Pelini, Mrs. M. Barbagallo fieldworkers; Mrs. A. Bossant, K. Guichardet, J.-T. Iltis, C. Martel, E. Quinteiro, neuropsychologists; Mrs. K. Supernant, A. Boudier the SEPAGES data managers; Mrs D. Nakiwala for thyroid hormones data cleaning; the staff from Grenoble Center for Clinical Investigation (CIC): Prof. J.-L. Cracowski, Dr. C. Cracowski, Dr. E. Hodaj, Mrs. D. Abry, Mr. N. Gonnet and Mrs. A. Tournier. A warm thank you also to Dr. M. Althuser, Dr. F. Camus-Chauvet, Mr. P. Dusonchet, Mrs. S. Dusonchet, Mrs. A. Royannais, Dr. D. Tournadre, Mr. P. Viossat, and clinicians from Grenoble University Hospital for their support in the recruitment of the study volunteers. We also thank Mrs. S. Caraby, Mrs. J. Dujourdil, and all midwives from the four maternity wards of the Grenoble urban areas. SEPAGES data are stored thanks to the Inserm RE-CO-NAI platform, funded by the Commissariat Général à l’Investissement, with the implication of Sophiede Visme (Inserm DSI). Many thanks to Dr. M.A. Charles, RE-CO-NAI coordinator, for her support. Finally, and importantly, we would like to express our sincere thanks to the participants of the Sepages study.

## Author contributions

Sandra Rjeschni: Data curation; Formal analysis; Methodology; Software; Visualization; Writing—original draft.

Ariane Guilbert: Writing—review & editing; Visualization.

Fanny Trecourt: Data curation, Conceptualization; Formal analysis, Visualization. Writing—review & editing.

Sarah Lyon-Caen: Investigation; Writing—review & editing.

Sam Bayat: Investigation; Writing—review & editing.

Rémy Slama: Conceptualization, funding acquisition, Investigation, Writing—review & editing.

Anne Boudier: Data curation; Writing—review & editing.

Patrice Faure: Assessment of thyroid hormones—review & editing.

Benoît Chovelon: Assessment of iodine - review & editing.

Christelle Corne: Assessment of thyroid hormones—review & editing.

Anne Sophie Gauchez: Assessment of thyroid hormones—review & editing.

Dorra Guergour: Assessment of thyroid hormones—review & editing.

Claire Philippat: Conceptualization, Data curation, Funding acquisition, Investigation, Methodology, Project administration, Resources, Supervision, Writing—review & editing.

Johanna Lepeule: Conceptualization, Data curation, Funding acquisition, Investigation, Methodology, Project administration, Resources, Supervision, Writing—review & editing.

## Data availability

The data used in this study can only be provided upon reasonable request after approval by the SEPAGES steering committee.

## Supplementary Material


